# MetaPSICOV: combining coevolution methods for accurate prediction of contacts and long range hydrogen bonding in proteins

**DOI:** 10.1093/bioinformatics/btu791

**Published:** 2014-11-26

**Authors:** David T. Jones, Tanya Singh, Tomasz Kosciolek, Stuart Tetchner

**Affiliations:** Bioinformatics Group, Department of Computer Science, University College London, London WC1E 6BT, UK

## Abstract

**Motivation:** Recent developments of statistical techniques to infer direct evolutionary couplings between residue pairs have rendered covariation-based contact prediction a viable means for accurate 3D modelling of proteins, with no information other than the sequence required. To extend the usefulness of contact prediction, we have designed a new meta-predictor (MetaPSICOV) which combines three distinct approaches for inferring covariation signals from multiple sequence alignments, considers a broad range of other sequence-derived features and, uniquely, a range of metrics which describe both the local and global quality of the input multiple sequence alignment. Finally, we use a two-stage predictor, where the second stage filters the output of the first stage. This two-stage predictor is additionally evaluated on its ability to accurately predict the long range network of hydrogen bonds, including correctly assigning the donor and acceptor residues.

**Results:** Using the original PSICOV benchmark set of 150 protein families, MetaPSICOV achieves a mean precision of 0.54 for top-*L* predicted long range contacts—around 60% higher than PSICOV, and around 40% better than CCMpred. In *de novo* protein structure prediction using FRAGFOLD, MetaPSICOV is able to improve the TM-scores of models by a median of 0.05 compared with PSICOV. Lastly, for predicting long range hydrogen bonding, MetaPSICOV-HB achieves a precision of 0.69 for the top-*L*/10 hydrogen bonds compared with just 0.26 for the baseline MetaPSICOV.

**Availability and implementation:** MetaPSICOV is available as a freely available web server at http://bioinf.cs.ucl.ac.uk/MetaPSICOV. Raw data (predicted contact lists and 3D models) and source code can be downloaded from http://bioinf.cs.ucl.ac.uk/downloads/MetaPSICOV.

**Contact:** d.t.jones@ucl.ac.uk

**Supplementary information:** Supplementary data are available at *Bioinformatics* online.

## 1 Introduction

Determining protein structure from its amino acid sequence remains an unsolved problem within bioinformatics (Dill and MacCallum, 2012). In order to make this notoriously complex goal more tractable, one simplification which can be made is to reduce the problem to identifying pairs of interacting residue pairs within the native structure, termed ‘contacts’. For many years, accurate contact prediction was hampered by the effects of the relatedness of homologous sequences and the inability to extract correct residue pairings from vastly intertwined networks of contacts (de Juan *et al.*, 2013). Recently, there has been considerable progress in reducing the effects of these two sources of noise, allowing for superior prediction accuracy (Taylor *et al.*, 2013).

Current methods for contact prediction use large multiple sequence alignments to identify interacting residues through correlated mutation analysis. Extracting contacts using correlated mutation analysis exploits the observation that within protein structures, interacting residue pairs are under evolutionary pressure to maintain their interaction (Altschuh *et al.*, 1987; Neher, 1994; Poon and Chao, 2005). In the event that one residue of such an interacting pair mutates, the effect of the mutation can be accommodated by the ‘correlated’ mutation of its interacting partner. Therefore, by identifying positions within a multiple sequence alignment which appear to coevolve, it is possible to infer that these positions are in close proximity in the native protein structure.

Contact maps derived from correlated mutation analyses are now of sufficient quality to enable accurate *de novo* structural modelling of both globular (Kosciolek and Jones, 2014; Marks *et al.*, 2011; Michel *et al.*, 2014) and membrane proteins (Hopf *et al.*, 2012; Nugent and Jones, 2012), as well as identifying residues at the interface of protein complexes (Ovchinnikov *et al.*, 2014; Weigt *et al.*, 2009). Whilst contacts are clearly useful for constraining modelling procedures, even small numbers of incorrect predictions are drastically detrimental to the modelling process (Konopka *et al.*, 2014). Despite considerable progress in the accuracy of predicted contacts from sequence, further increased precision will allow for more accurate models to be produced (Michel *et al.*, 2014).

It has been shown recently that the incorporation of two different coevolution methods, based on distinct underlying principles, is able to increase the accuracy of contact prediction (Skwark *et al.*, 2013). In this study, we develop MetaPSICOV, a hybrid method that combines a classical neural network-based contact prediction method with three different coevolution methods, in order to improve the accuracy of predicted contacts from multiple sequence alignments. An additional aspect of MetaPSICOV is to modulate predictions according to both the local and global quality of the multiple sequence alignment. Thus for alignments that have little depth, either in local regions or over the entire length of the target protein, MetaPSICOV can effectively down-weight the optimal contributions that should be made by coevolution compared with more traditional features such as predicted secondary structure and solvent accessibility. This means that for poor alignments, MetaPSICOV will behave more like a standard machine learning-based contact prediction method, whereas for deep high-quality alignments more emphasis will be placed on coevolution signals.

## 2 Methods

### 2.1 Datasets for training and benchmarking

The benchmark test set for this study is the original PSICOV test set of 150 single domain monomeric proteins with the same alignments as made publicly available back in 2011 (Jones *et al.*, 2012). To ensure full comparability with earlier work, no attempt was made to extend the alignments using more up to date sequence data banks.

The neural network components of MetaPSICOV were trained on a set of 624 highly resolved protein chains with no overlap to the test set. To start with, we took a non-redundant (percent sequence identity <25%) set of 1780 protein chains from PDB (Berman *et al.*, 2000) with resolution ≤1.5 Å and chain lengths between 50 and 500 residues. Overlapping proteins between the original PSICOV test set and the initial training set were identified by using HHSEARCH (Söding, 2005) to align test set sequences with training set HMMs. Alignments and HMMs for the training set were calculated using three iterations of HHBLITS (Remmert *et al.*, 2012) with an *E*-value threshold of 10^−3^ and the March 2013 release of the UNIPROT20 HMM library. The alignments were stored in both PSICOV (Jones *et al.*, 2012) native and A3M formats. Any chain in the initial training set matching a test set sequence with an HHSEARCH E-value <10^−^^3^ was excluded, leaving a final training and validation set of 624 chains (Supplementary Table S1).

To investigate performance of MetaPSICOV on smaller protein families, we collected an additional set of PDB chains with no overlap with the training set (HHSEARCH *E*-value >1.0), resolution ≤2.0 Å and chain lengths between 50 and 400 residues. For these protein chains, alignments were generated with HHBLITS as described above, and chains with between 50 and 500 homologous sequences were selected as the second test set. This second test set comprised 434 chains (Supplementary Table S2).

Three separate coevolution calculations on the alignments were carried out using PSICOV v2.1, mean field DCA as implemented in the FreeContact package (Kaján *et al.*, 2014) and CCMpred (Seemayer *et al.*, 2014). The same alignments were used in each case, in PSICOV format for PSICOV and FreeContact and A3M format for CCMpred.

In addition to the coevolution features, a number of other simple statistics are computed from the same alignments using a C program (alnstats). These statistics are listed in the feature set section below, and are divided into single alignment column-based statistics (e.g. the amino acid frequencies in each column) and pairwise column statistics (e.g. mutual information scores between each pair of columns).

### 2.2 MetaPSICOV input features

A total of 672 input features are used in the first stage classifier to predict the likelihood of residue *i* and *j* being in contact. As a starting point for input features, we considered the feature set used in the SVMcon method (Cheng and Baldi, 2007). Here, in both training and predicting, three windows are defined: one nine residue window centred at position *i*, one nine-residue window at position *j* and a central window of five residues at the midpoint *½*(*i* + *j*). At every position in each window, 21 features provide information on the amino acid composition in alignment columns *i*, *j* and *½*(*i* + *j*), counting gaps as the 21st amino acid type. The next four column features are the probabilities of helix, strand and coil states, and a predicted solvent accessibility in the range 0–1 (0 being completely buried and 1 being fully exposed). The secondary structure probabilities are derived from PSIPRED (Jones, 1999), and the solvent exposure from a derivative of PSIPRED called SOLVPRED. The final two column features are the Shannon entropy (−*∑k p_k_* log *p_k_*) of the alignment column and an extra input to indicate missing data, i.e. where the window exceeds the limits of the sequence.

The next set of input features (six in total) provide coevolution information for each pair of alignment columns, and are as follows: mutual information, normalized mutual information (Dunn *et al.*, 2008), mean contact potential, PSICOV score, mfDCA score and CCMpred score. The mean contact potential is computed by averaging contact potential terms (Betancourt and Thirumalai, 1999; Miyazawa and Jernigan, 1985) across the two alignment columns.

To encode the sequence separation |*i* − *j*|, 16 features are used to represent values in the following ranges: |*i* − *j*| < 5, |*i* − *j*| = {5,6,…,13}, 14 ≤ |*i* − *j*| < 18, 18 ≤ |*i* − *j*| < 23, 23 ≤ |*i* − *j*| < 28, 28 ≤ |*i* − *j*| < 38, 38 ≤ |*i* − *j*| < 48, |*i* − *j*| ≥ 48.

The remaining features are derived from the whole alignment and do not depend on the window locations: global alignment amino acid composition (21 features including gap composition), global fractions of predicted secondary structure states (three features), global average-predicted solvent exposure, log sequence length, log number of sequences in the alignment, log effective number of sequences in the alignment and global average Shannon entropy. Effective sequence counts were calculated by clustering protein sequences with a 62% sequence identity threshold, and then assigning equal fractional weights (*w_i_ = *1/*n*) to sequences in each cluster, where *n* is the cluster size. Effective sequence counts are then calculated as *N*_eff_ = ∑*w_i_*. A summary table of the features used is given in Supplementary Table S3.

For the second stage classifier, which correlates the outputs of the first stage classifier, a total of 731 input features are used. In this case, two windows of 11 alignment columns are defined at location *i,* *j*, though the central window is omitted in this case. The same column features defined for the first stage classifier are also used for the two windows in the second stage. In addition the same sequence separation features are included. The remaining 121 features comprised an 11 × 11 matrix of values, centred at location *i,* *j* which are taken from the relevant outputs of the first stage (summarized in Supplementary Table S3). This matrix therefore represents a 2D (square) window on the first stage predicted contact map, and thus allows a refined contact map to be generated by taking into account correlations between nearby predicted contacts from the first stage network. This second stage also allows us to predict other kinds of inter-residue interactions, e.g. main chain hydrogen bonding, as described later.

### 2.3 Neural network architectures and training procedure

Both first and second stage classifiers are classic feed-forward neural networks, with 55 hidden units and a single output unit. To train these very large networks, alternate rounds of offline and online training are carried out until no further improvement in accuracy is obtained on a separate validation set, which comprises 10% of the original training data.

The classification of residues as contacting (true positive) or non-contacting (true negative) was made by calculating Cβ–Cβ distances in the protein chains, with different thresholds. For glycine residues, the Cα position was used in place of the Cβ. In final benchmarking, only the usual threshold of 8 Å was used, but in training we used five different contact distance thresholds: 6, 7.5, 8, 8.5 and 10 Å. An additional criterion that was used was to set a threshold distance of 8 Å, but then only consider negatives where the distance exceeded a second threshold of 11 Å. In this latter case, residue pairs with an intermediate distance were excluded from the training process. Each of these six thresholds was used to generate a separate neural net and in making a prediction, the outputs of the six networks are combined by averaging. As Cβ–Cβ contacts are symmetric, for each *L* residue protein chain in the training set, ½*L*(*L* − 1) residue pairs are considered in testing and training.

The training data are obviously highly unbalanced given that only around 5% of residue pairs make contacts in a typical protein fold. However, we find that subsequent calibration of the network outputs is a better way of handling this bias than either undersampling negative cases or oversampling the positive cases. Once the network training is complete, the outputs can be transformed to estimated Positive Predictive Values (PPVs) by a log-linear function:

PPV=a log(bx+c),

where *a*, *b* and *c* are adjustable parameters obtained by least-squares fitting to the observed PPVs when the training data are re-presented to the networks and binned according to neural network output score (*x*).

### 2.4 MetaPSICOV-HB

By a small modification to the second stage classifier and the training scheme, it is straightforward to develop an additional predictor which can specifically predict main chain hydrogen bonding patterns rather than generic residue–residue contacts. In this case, positive cases will be residue pairs where the first residue acts as donor for a backbone hydrogen bond (N–H···O = C) to the other. In this case, the relevant contact is directional and so the resulting contact map can no longer be assumed to be symmetric. In antiparallel sheets, of course, a symmetric pair of contacts can occur, as a bridged pair of residues will be involved in two hydrogen bonds going in opposite directions.

In training and testing, we arbitrarily assign the donor role to residue *i* and the acceptor role to residue *j*, and a true positive is only considered where the amino nitrogen in *i* is within 3.5 Å of the carbonyl oxygen in *j*. No attempt was made to reconstruct the amide hydrogen positions as for main chain hydrogen bonds in highly resolved proteins, the heavy atom distance criterion is generally sufficient on its own to determine whether an energetically favourable hydrogen bond is formed. Short range (sequence separation <5) hydrogen bonds are not considered because these mostly relate to alpha helices and can be trivially inferred from secondary structure prediction alone, and so the sequence separation binary input for the |*i* − *j*| < 5 case was replaced by a binary feature which simply indicates pairs where *i* > *j*, which is needed because pairs *i,* *j* and *j,* *i* can no longer be considered equivalent.

### 2.5 Folding simulations

To evaluate the usefulness of MetaPSICOV contacts in *de novo* protein structure prediction, we carried out a benchmark on contact-assisted fragment assembly using FRAGFOLD (Jones *et al.*, 2005). Details of the benchmark have already been described (Kosciolek and Jones, 2014), where we evaluated the usefulness of PSICOV contacts in assisting *de novo* protein structure prediction. Two slight modifications were made here. First, a slightly revised FRAGFOLD energy function is used, with short range (|*i* − *j*| < 23) and long range contacts treated as two separate equally weighted energy terms. Second, to select a single representative model from each ensemble, the model with the lowest total FRAGFOLD energy was selected. This is a more conservative selection procedure than our previous study, where we took the best model from the five lowest energy models, but this more closely represents the way in which models would be selected in practice.

## 3 Results and discussion

**Fig. 1.**
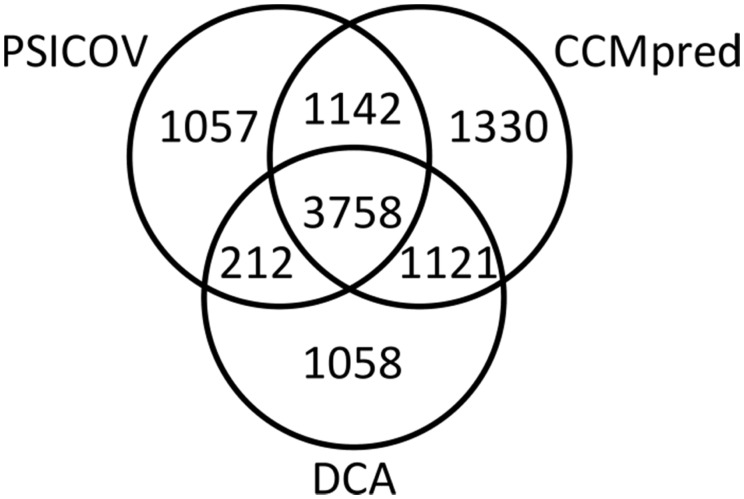
Distribution of predictions for 9678 observed true contacts, as predicted by PSICOV, DCA and CCMpred. Data are the top *L*/2 correct contact predictions from each method (for sequence separation >=5 residues), for a set of 150 Pfam families (Jones *et al.*, 2012)

The accuracies of MetaPSICOV and the component methods were evaluated on the widely-used benchmark dataset of 150 proteins used to test the original PSICOV algorithm (Jones *et al.*, 2012). Figure 1 shows the relative overlap between the three component coevolution methods applied to this set, which clearly demonstrates the value of a three-way consensus, with at least 10% of correct contacts (13% in the case of CCMpred) being unique to each method.

The analysis of results is divided into two parts, based on the sequence separation of predicted pairs, as contact predictions between residue pairs lying far apart in the sequence are more difficult to predict than the ones closer in sequence separation. With this in mind, mean precision scores are given at sequence separation ≥5 and ≥23 residues, respectively. Two residues are considered to be in contact with each other if the Cβ–Cβ distance is <8 Å, which is in accordance with the standard assessment procedure introduced in the Critical Assessment of protein Structure Prediction experiment (Ezkurdia *et al.*, 2009). Table 1 depicts the mean precision values obtained for different methods including PSICOV, DCA, CCMpred and both stages of MetaPSICOV for the top-*L*/10, top-*L*/5, top-*L*/2 and top-*L* contacts where *L* is the length of the target protein, the thresholds used commonly in contact prediction assessment methods, for sequence separation ≥5 and ≥23 simultaneously (Ezkurdia *et al.*, 2009).

**Table 1.** Mean precision values for the top-*L*, top-*L*/2, top-*L*/5 and top-*L*/10 contacts, *L* = length of target protein, at different sequence separation ranges *i* − *j* ≥ 5 and *i* − *j* ≥ 23, where the Cβ–Cβ distance <8 Å

	[*i* − *j*] ≥ 5				[*i* − *j*] ≥ 23			
	*L*	*L*/2	*L*/5	*L*/10	*L*	*L*/2	*L*/5	*L*/10
Net only	0.37	0.41	0.52	0.66	0.25	0.32	0.40	0.43
PSICOV	0.45	0.60	0.73	0.79	0.34	0.48	0.65	0.73
DCA	0.45	0.56	0.66	0.72	0.36	0.49	0.64	0.71
CCMpred	0.52	0.67	0.77	0.81	0.39	0.55	0.71	0.76
Consensus only	0.51	0.65	0.77	0.81	0.39	0.54	0.70	0.77
PconsC	0.56	0.69	0.77	0.79	0.46	0.62	0.75	0.80
MetaPSICOV (stage 1)	0.66	0.79	0.90	0.95	0.47	0.63	0.78	0.85
MetaPSICOV (stage 2)	0.71	0.83	0.91	0.94	0.54	0.70	0.83	0.88

To allow the relative contributions of individual components to be gauged, various subsets of features were also evaluated. The main subsets are shown in Table 1, and some additional subsets in Supplementary Table S4.

The worst performing subset is unsurprisingly the subset excluding the three coevolution methods, though the true positive rate, even in this case, is far from negligible. One surprise is that a pure consensus (‘Consensus only’) of the coevolution methods performs no better than the best single method (CCMpred). This suggests that the additional features in MetaPSICOV are vital in correctly deciding the relative weighting to assign to the different coevolution inputs. A network without any of the coevolution inputs (‘Net only’) performs very poorly on long range contacts, though when shorter range contacts are included, a reasonable level of prediction accuracy is obtained.

The seven different combinations of coevolution methods are evaluated in Supplementary Table S4, where it can be seen that a combination of all three methods performs optimally.

**Fig. 2.**
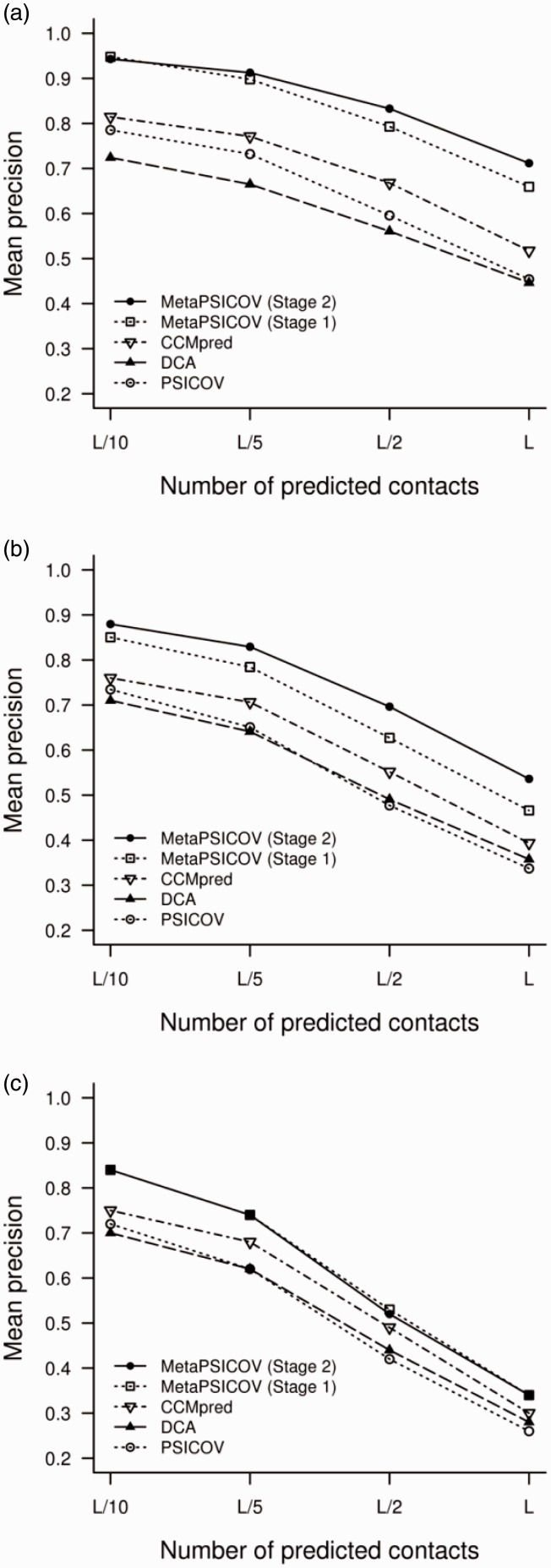
Mean precision of contact prediction against increasing number of predicted pairs for (**a**) sequence separation ≥5, (**b**) for sequence separation ≥23 and (**c**) for sequence separation ≥23 with redundant contact predictions excluded (see text for details)

**Fig. 3.**
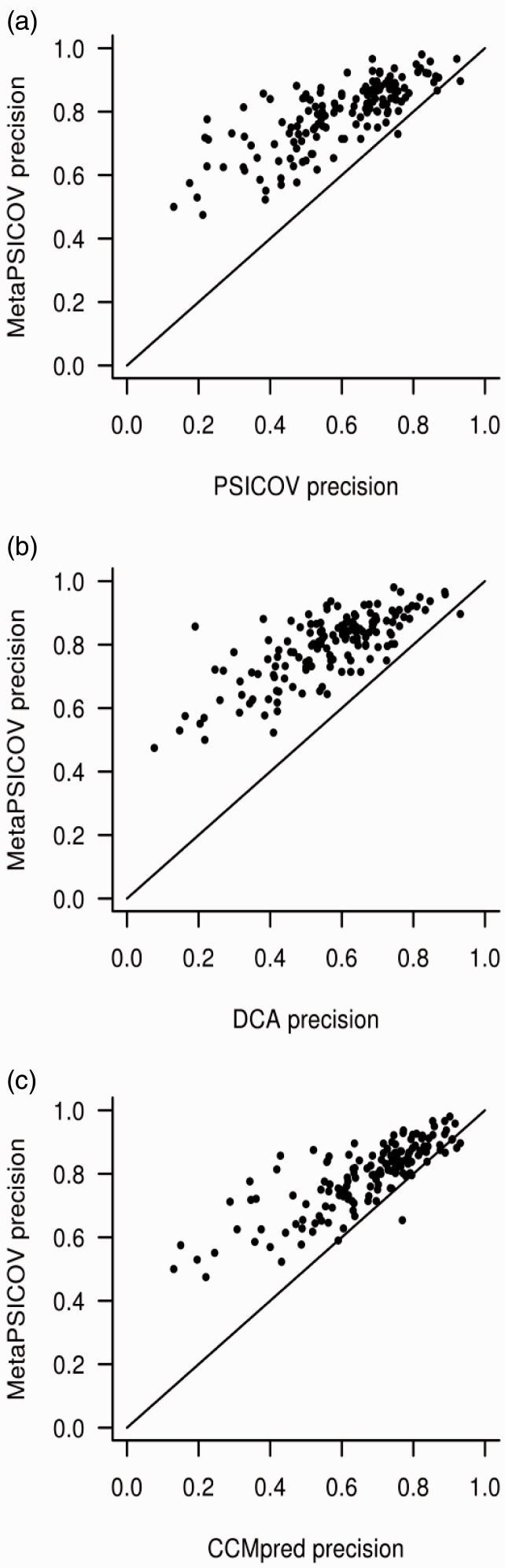
MetaPSICOV top-*L*/2 precision plotted against (**a**) PSICOV precision, (**b**) DCA precision and (**c**) CCMpred precision for sequence separation ≥5 (line *x* = *y* shown for reference)

Figure 2 shows the overall performance of MetaPSICOV in terms of its mean precision, both after the first stage and the second filtering stage. For comparison, results for CCMpred (Seemayer *et al.*, 2014), PSICOV (Jones *et al.*, 2012) and DCA (Marks *et al.*, 2011) are also shown. Clearly, both stages of MetaPSICOV significantly outperform the other methods at both sequence separation ranges and all four contact subsets. To see this in more detail, Figure 3 shows a scatter plot between the mean *L*/2 precision against the three component coevolution methods. Again, MetaPSICOV shows substantially higher accuracy precisions than any of the other tested component methods in almost every case.

To check for possible bias from very distant relationships between domains in the training set and domains in the testing set that could not be detected by HMM–HMM comparison, we recalculated the performance of MetaPSICOV on a subset of the PSICOV training set with no domain superfamilies in common with the training set, according to the CATH structure-based classification (release 4.0; Orengo *et al.*, 1997). This additional constraint reduces the PSICOV test set from 150 to 101 proteins (see Supplementary Table S5a). Using a standard *t* test, for all mean precision values calculated for MetaPSICOV, no significant differences were observed after further structure-based cross-validation (see Supplementary Tables S5b and S5c).

One aspect of contact prediction assessment that is typically ignored is the relative redundancy between different predicted contact maps. Let us suppose that we already know that there is a contact between residues *i* and *j* in a particular protein. Without even looking at the structure or making a calculation we can guess that residues in the immediate sequence neighbourhood of this contacting pair (*i* + 1, *j* − 1 for example) are also likely to be in contact. Of course not all of these contacts in the immediate neighbourhood will be correct, and the exact pattern will depend on whether the chain segments are in beta sheets and then in a parallel or antiparallel conformation. Nevertheless, it is fair to say that pairs of contacts that are close together along the chain can be considered somewhat redundant in terms of predicting the overall chain fold.

To evaluate the effects of redundancy on our comparison of methods, we recalculated the benchmark results after filtering out any lower scoring predicted contacts that are immediately adjacent (*i* ± 1, *j* ± 1) to any higher scoring predictions. Filtering was applied to both correct and incorrect contacts; therefore, methods are on the one hand not credited for making multiple adjacent correct contact predictions, and on the other hand, not multiply penalized for making adjacent incorrect predictions. This is admittedly a very conservative benchmarking strategy, as neighbouring contacts clearly have at least some additional value in constraining the chain fold, but their value is clearly much less than unique long range contacts that are correctly predicted. Figure 2c shows the impact of excluding multiple predicted adjacent contacts. Firstly, it is clear that all the methods are much more similar in terms of overall performance. Furthermore, at least some of the differences between the component methods derive from more redundant contacts. MetaPSICOV still outperforms the other methods, albeit with a reduced margin, but there is now no difference between the first and second stage classifiers. This is entirely unsurprising, as the whole point of the second stage network is to analyze the neighbourhood of predicted contacts from the first stage network in order to add missed contacts or remove spurious predictions.

**Fig. 4.**
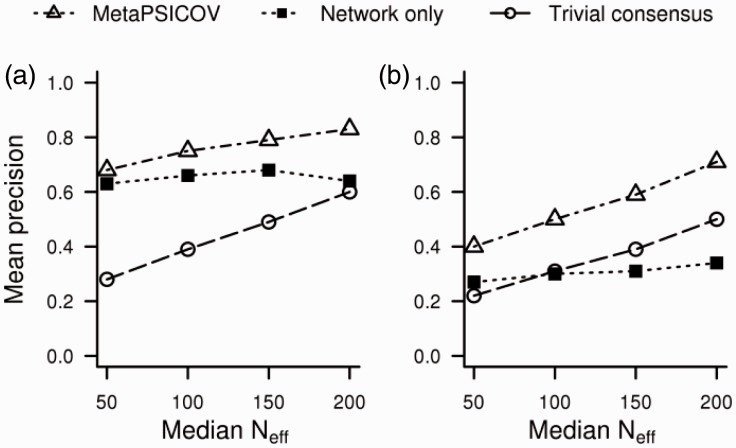
All contacts (**a**) and long-range contacts (**b**) top-*L*/10 mean precision for alignments with varying numbers of effective sequences (*N*_eff_)

As a final test, we tested MetaPSICOV on a set of target proteins with much smaller sequence families than the original PSICOV test set (see Supplementary Table S2). Figure 4 summarizes the results for MetaPSICOV compared with the trivial consensus of covariation methods and the traditional contact prediction (network-only) results (see Supplementary Fig. S1 for related plots of top *L*/5, *L*/2 and *L* contacts). Here it is evident that, as expected, MetaPSICOV relies heavily on the traditional contact prediction features where few sequences are available, but steadily increases weight on the covariation data as the number of effective sequences approaches 200. Another interesting point is that performance of MetaPSICOV with 200 effective sequences is roughly the same as the best individual covariation-based method with a median effective sequence count of 621 (the median for the original PSICOV test set). Thus, MetaPSICOV is able to extend the usefulness of covariation-based contact prediction to significantly smaller families than those that have been the focus of earlier studies.

It is of course of interest to compare MetaPSICOV with the recently published coevolution-based contact metapredictor, PconsC. Although the general idea of combining multiple coevolution methods is the same in both cases, the philosophy behind the ways in which variation in alignment quality is taken into account is substantially different. In the case of PconsC, to achieve maximum performance a set of eight different alignments are required, from two different alignment methods and four parameters settings in each case. In the case of MetaPSICOV, however, a single alignment is used, but is supplemented by an array of features which describe the local and global quality of the given alignment. As both methods make use of the same testing set of 150 proteins used in testing PSICOV, it is also possible to make at least a rough quantitative comparison between them. One slight complication is that PconsC does not make use of the original PSICOV alignments, but instead uses several combined alignments that clearly will include more up-to-date sequence data. That being said, MetaPSICOV still appears to perform well in comparison to PconsC, as can be seen in Table 1.

The fact that MetaPSICOV can achieve better results than PconsC without requiring sets of alignments to be generated from different methods and different runtime parameters is clearly advantageous in terms of convenience and reduced computation. This also possibly insulates MetaPSICOV from systematic changes in both the underlying sequence data banks and the alignment methods as time goes on. However, it is certainly worth considering that MetaPSICOV might be further improved by also being trained on a range of alignments, and this is something that we may explore in the future.

The mean precision scores for MetaPSICOV-HB and a naïve method based on the standard MetaPSICOV algorithm for the top-*L*/10 contacts is reported in Table 2. The top-*L*/10 contacts are chosen in this case as there are significantly fewer long range hydrogen bonds in proteins compared with general long range contacts. For the naïve method, any top-*L*/10 contact predicted by MetaPSICOV between residues in predicted beta strands is assumed to be a donor/acceptor pair, but with the donor–acceptor labelling being ignored. For MetaPSICOV-HB, however, only correctly labelled donor–acceptor pairs are counted as true positives. Table 2 clearly shows the added value that MetaPSICOV-HB gives over the standard method when predicting the hydrogen bonding networks in proteins, with a mean precision value 0.69 for long range hydrogen bonds compared to 0.26 for the naïve method. This additional information on the directions of hydrogen-bonded residue pairs could be invaluable in improving the quality of models produced by *de novo* methods, though finding the optimal way to use this information will take some effort.

**Table 2.** Mean precision values for the Top-*L*/10 predicted main chain hydrogen bonds at different sequence separation ranges, where residue *i* donates a main chain hydrogen bond to residue *j* (N–H···O = C distance <3.5 Å)

	[*i* − *j*] ≥ 5	[*i* − *j*] ≥ 23
	*L*/10	*L*/10
MetaPSICOV	0.43	0.26
MetaPSICOV-HB	0.81	0.69

The 17 mainly helical targets with fewer than *L*/10 true hydrogen bonds were omitted from this analysis.

The last part of this study involves evaluating the use of MetaPSICOV contact constraints for improving *de novo* protein structure models. For each of the 150 benchmark proteins, 20 models were generated by FRAGFOLD using three different predicted contact sets: PSICOV, MetaPSICOV stage 1 and MetaPSICOV stage 2 contacts (9000 models in total). To ensure that all useful contact information was being used, each contact list was cut off at a precision threshold of 0.5, i.e. all the contacts used were estimated to have a >0.5 probability of being correct.

**Fig. 5.**
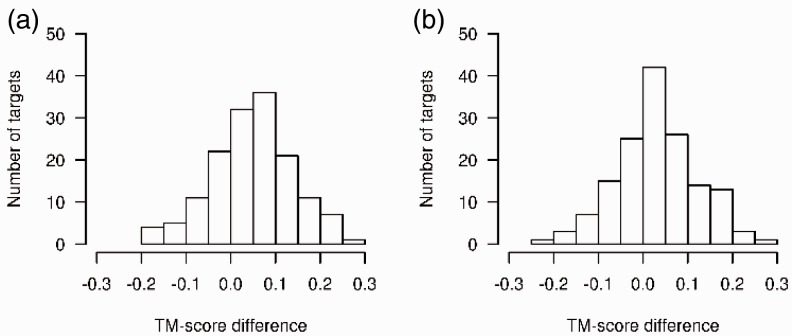
Differences in mean TM-scores for the 150 benchmark proteins obtained by FRAGFOLD using (**a**) MetaPSICOV stage 1 contacts and (**b**) MetaPSICOV stage 2 contacts compared with PSICOV contacts

Without any contact constraints, the median TM-score for FRAGFOLD without contact constraints is 0.27, compared with 0.41 for PSICOV, 0.46 for the first stage MetaPSICOV contacts and 0.43 for the second stage contacts. Figure 5 shows the distributions of TM-score differences between the PSICOV representative models and the MetaPSICOV models from each target ensemble. Scatter plots for each comparison along with the underlying data are shown in Supplementary Figure S2 and Supplementary Table S6. For 108 targets out of 150, the first stage MetaPSICOV contacts produce better models than PSICOV with a median improvement of 0.05 TM score units. For 40 targets, however, the improvement is quite substantial with an improvement of at least 0.1 TM units. Surprisingly, given that more correct long range contacts are included in the second stage contact lists, results are slightly worse than those from the first stage, with only 99 targets showing an improvement, and a median improvement of only 0.02 TM units. Although this looks puzzling at first glance, the explanation is almost certainly down to the same redundancy issue that has been described previously. Suppose we were to predict the full set of contacts between a pair of hydrogen-bonded beta strands, but just two contacts between a pair of helices elsewhere in the structure. The additional contacts along the strands are not particularly informative as most derive purely from the regular hydrogen bonding pattern. In this case, therefore, correctly modelling the beta strand pairing might satisfy five times as many constraints as correctly modelling the pair of helices, even though the helices may contribute far more to the overall model TM score. Consequently, the global optimization procedure is likely to be strongly biased towards satisfying the beta strand constraints rather than the constraints between the helices in this hypothetical case. Note that this is only an issue because the contact constraints are probabilistic in nature. If all the constraints were real, then it is obvious that the best possible model would then result from simply satisfying all of the contacts.

By analyzing the test set by fold class (α, αβ and β) we see some support for this hypothesis. Ignoring the 10 targets with few secondary structures, for the 16 all-α targets, a positive median TM-score difference of 0.04 is indeed observed between the first and second stage results, whereas for the 93 αβ and α + β targets a median difference of −0.05 is observed. For the 31 all-β targets no difference is observed.

Although the sample sizes make firm conclusions impossible, these results do suggest that appropriate balancing between α-helix and β-sheet contacts is needed to exploit the second stage predicted contacts properly. Not only do short range contacts need to be separated from long range contacts (as we already do), but the redundancy in 3D constraints between neighbouring contacts also needs to be considered in an ideal weighting scheme. How this should best be done is as yet unclear. It would obviously be easy to exclude neighbouring contacts as shown in Figure 2c, but then the additional information would just be lost. A better approach may be to cluster the contacts into groups, according to secondary structure and sequence separation, perhaps, and then apply a suitable weighting to each cluster.

## 

## 4 Conclusion

Although recent developments in algorithms to determine coevolution patterns in large multiple sequence alignments have created a lot of excitement in structural bioinformatics, coevolution alone is clearly not enough to derive all of the likely contacts in a protein structure. Although it is an impressive feat to deduce distance constraints purely from a statistical analysis of a multiple sequence alignment, it is evident that other sources of information can also contribute to producing more accurate-predicted contact maps. It makes no sense to limit one’s analysis solely to one source of information and ignore other sources, albeit sources that are weaker predictors in their own right.

MetaPSICOV is an effective hybrid of a fairly standard machine learning-based contact prediction method with three state-of-the-art coevolution-based methods. The neural network is able to make a balanced decision between the coevolution signals and the generic structural features that provide orthogonal information on the likelihood of residue pairs making a contact. For target proteins with poor quality or sparse multiple sequence alignments, the appropriate strategy is to de-emphasize coevolution in favour of generic structural features, but where sufficient homologous sequences are available, the overwhelming value of coevolution is clear.

Compared with the best single coevolution method, MetaPSICOV achieves a precision that is 38% higher than the best individual coevolution method (CCMpred) for the top-*L* long range contacts. Furthermore, the mean precision for MetaPSICOV of 0.54 indicates that correct predicted contacts are more likely than incorrect contacts, which is an important performance threshold in terms of accurate 3D model building from predicted contacts (Hopf *et al.*, 2012; Kosciolek and Jones, 2014; Marks *et al.*, 2011; Michel *et al.*, 2014; Nugent and Jones, 2012).

One of the more interesting observations has been the apparent effect of redundancy in predicted contacts. By constructing a two-stage classifier it is possible to produce a more accurate contact predictor, but at the expense of biasing the distribution of contacts to regions of the protein where adjacent contacts are made (beta sheets). Although benchmarks clearly show that more correct contacts are predicted by the second stage of the MetaPSICOV, the added bias in fact results in generally poorer 3D models when the contacts are used for *de novo* structure prediction using FRAGFOLD. Better contact constraint scoring functions, which more evenly weight contact constraints according to both sequence separation and neighbourhood density, are needed to deal with this problem.

Comparing MetaPSICOV to PconsC, another published coevolution-based consensus contact predictor, MetaPSICOV, evidently outperforms the earlier method on the same benchmark set. However, there are interesting aspects of PconsC that are not currently considered within the MetaPSICOV feature set, e.g. features from sampling more than one sequence alignment from different methods or alignment parameters. Consequently, even better hybrid methods may be envisaged by considering even larger feature spaces.

Finally we show that it is possible to apply MetaPSICOV to predicting not just contacting residue pairs, but explicitly identifying donor/acceptor pairings in beta sheets. By combining the accurate prediction of side chain contacts with this much more discriminating structural information, it should be possible to greatly enhance the quality of *de novo* modelling in beta-sheet rich target proteins.

## Funding

This study was funded by the Biotechnology and Biological Sciences Research Council [grant number BB/L018330/1; T.S. and D.T.J.] and the Wellcome Trust [grant number 096624/Z/11/Z to T.K. and grant number 096622/Z/11/Z to S.T.].

*Conflict of Interest*: none declared.

## Supplementary Material

Supplementary Data
