# Supplementary material for: MetaPSICOV: combining coevolution methods for accurate prediction of contacts and long range hydrogen bonding in proteins
Source: Bioinformatics. 2014 Nov 26;31(7):999–1006. doi: 10.1093/bioinformatics/btu791 (PMC4382908; doi:10.1093/bioinformatics/btu791)
Supplement: Supplementary Data [file supp_31_7_999__index.html]

MetaPSICOV: combining coevolution methods for accurate prediction of contacts and long range hydrogen bonding in proteins — MetaPSICOV: combining coevolution methods for accurate prediction of contacts and long range hydrogen bonding in proteins — Supplementary Data 

# MetaPSICOV: combining coevolution methods for accurate prediction of contacts and long range hydrogen bonding in proteins

## Supplementary Data

files

**Files in this Data Supplement:**

- Supplementary Data - pdf file
