# Supplementary material for: MetaPSICOV: combining coevolution methods for accurate prediction of contacts and long range hydrogen bonding in proteins
Source: Bioinformatics. 2014 Nov 26;31(7):999–1006. doi: 10.1093/bioinformatics/btu791 (PMC4382908; doi:10.1093/bioinformatics/btu791)

Supplementary Table S1

a) List of 561 PDB Chains used in the MetaPSICOV training set.

1bgfA 1bx7A 1c5eA 1dbfA 1e4mM 1e5kA 1e7lA 1eaqA 1eb6A 1f8eA 1fsgA 1g61A 1gk8I 1gk9A 1gqvA
1gu2A 1gveA 1gyxA 1h4gA 1hbnB 1hbnC 1hyoA 1i27A 1jcdA 1ji7A 1jniA 1k5cA 1ka1A 1kkoA 1kmtA
1kqpA 1kv0A 1kyfA 1l9lA 1lc5A 1lniA 1lwbA 1lyvA 1m1qA 1m2dA 1m55A 1mkkA 1mn8A 1munA 1muwA
1mxrA 1n8vA 1nkdA 1nqjA 1nuyA 1nz0A 1o2dA 1o4yA 1o7qA 1odmA 1oh4A 1ox0A 1p4cA 1pbjA 1q5yA
1q7lA 1q7lB 1qh4A 1qtwA 1qw9A 1r29A 1r85A 1ra0A 1rkuA 1sauA 1sjwA 1svfA 1t6uA 1tt8A 1u07A
1u7iA 1ucrA 1ucsA 1ukuA 1uz3A 1v05A 1vmfA 1vp8A 1vr7A 1vykA 1w0nA 1w1hA 1w66A 1wc2A 1wcwA
1wluA 1wn2A 1wpnA 1wzdA 1x6iA 1x6zA 1xbiA 1xd3A 1xdnA 1xmtA 1xqoA 1xubA 1yfqA 1yiiA 1z0nA
1zgkA 1zzkA 256bA 2aibA 2b0aA 2b3hA 2b82A 2b97A 2bcgG 2bkxA 2bmoA 2bmoB 2c0rA 2c1vA 2c2uA
2c4bA 2c60A 2carA 2cb8A 2ccvA 2cg7A 2ckkA 2covD 2cs7A 2dkoB 2dlbA 2ehzA 2endA 2erfA 2f22A
2fbaA 2fcjA 2fcwB 2fmaA 2fsqA 2g7sA 2gkeA 2gkpA 2glzA 2gsoA 2gudA 2h1vA 2h8eA 2halA 2hbaA
2hoxA 2hzcA 2i5vO 2iayA 2ic6A 2imsA 2ivyA 2j5yA 2j6bA 2j8bA 2jekA 2jliA 2nlvA 2nr7A 2nszA
2nxvA 2o37A 2o90A 2odiA 2odkA 2ofzA 2olmA 2ovgA 2ovjA 2oxgA 2oxgB 2p0nA 2p2sA 2p51A 2p8gA
2pieA 2pneA 2q52A 2qfaB 2qfeA 2qipA 2qjzA 2qngA 2qsbA 2r0xA 2r16A 2r2zA 2r31A 2r5oA 2r751
2ra9A 2rctA 2rdqA 2rfrA 2rh2A 2uvoA 2ux9A 2v89A 2v8tA 2v9vA 2ve8A 2vfrA 2vn6B 2vwsA 2w3gA
2w5qA 2w7aA 2wbqA 2wcjA 2wdsA 2wl1A 2wnpF 2wq4A 2x3mA 2x4kA 2x5yA 2xfdA 2xioA 2xjpA 2xmjA
2xomA 2xpwA 2xw6A 2y0oA 2y39A 2y5pA 2y6hA 2y9fA 2yh5A 2ylbA 2ylnA 2z4uA 2z5wA 2zexA 2znrA
3a02A 3a09A 3a6rA 3a9bA 3achA 3acxA 3agnA 3aiaA 3ak2A 3amrA 3ap9A 3awuA 3b79A 3bbbA 3bfqG
3bjeA 3bmzA 3bnjA 3bo6A 3boeA 3bqpA 3bwzA 3bxuA 3chbD 3chvA 3ci3A 3cijA 3cimA 3cjsB 3ckmA
3d32A 3d3bJ 3d4eA 3d9xA 3db7A 3e4gA 3e8tA 3eerA 3eo6A 3eyeA 3eyiA 3f40A 3f6vA 3f6yA 3f7eA
3f7xA 3f9xA 3fciA 3fdeA 3feaA 3fghA 3fgvA 3filA 3g0kA 3g16A 3g21A 3g4eA 3gbwA 3giuA 3gneA
3gveA 3gxhA 3gzrA 3h0nA 3h4tA 3h4xA 3h87A 3h87C 3hfoA 3hhtB 3hlxA 3hnxA 3hwuA 3hx8A 3hynA
3i4oA 3i6cA 3i7mA 3i94A 3ib5A 3ie4A 3iisM 3imkA 3ip0A 3isxA 3itfA 3ivvA 3ix3A 3jumA 3k5jA
3k67A 3k7iB 3ka8A 3kuuA 3kwrA 3l1eA 3l41A 3l46A 3l8wA 3ldcA 3ledA 3lftA 3lydA 3lzqA 3m0mA
3m3pA 3m5qA 3m7aA 3m9qA 3mabA 3maoA 3mmhA 3mqzA 3mwxA 3myxA 3n01A 3n4jA 3njnA 3no2A 3ns6A
3nvsA 3nwpA 3nytA 3nzlA 3o1nA 3o8mA 3oajA 3oblA 3od9A 3oruA 3ov5A 3oyvA 3p4hA 3pd7A 3pesA
3pfzA 3piwA 3pkvA 3pmcA 3pojA 3pplA 3q39A 3q3yA 3qc0A 3qdsA 3qhbA 3ql9A 3qooA 3qu3A 3r5tA
3rkgA 3robA 3rpcA 3rq9A 3rwnA 3s1yA 3s8sA 3s9xA 3seeA 3sk2A 3so6A 3sojA 3sovA 3su6A 3sukA
3sy1A 3t2cA 3t47A 3t49A 3t94A 3tm8A 3tnlA 3tvjA 3tysA 3u6gA 3u7qA 3u7zA 3u97A 3ub6A 3uciA
3uejA 3uhmA 3ur8A 3uxjA 3v39A 3v4kA 3vorA 3vqjA 3vrcA 3vubA 3vypA 3vz9B 3w5hA 3wg3A 3wkgA
3zfpA 3zhnA 3zk9A 3zn4A 3znvA 3zpyA 3zudA 3zzoA 3zzpA 4a02A 4a56A 4a8uA 4acjA 4afmA 4al0A
4annA 4attA 4au1A 4avsA 4b1mA 4b62A 4b9pA 4bfoA 4bgbA 4boqA 4bpsA 4brcA 4bt7A 4c45A 4cayB
4co8A 4d8bA 4di9A 4dqjA 4driB 4dt5A 4dwrA 4ebgA 4eguA 4emnA 4enfA 4ep4A 4espA 4euoA 4exkA
4f3jA 4f87A 4f8zA 4f98A 4fchA 4fglA 4fioA 4fkbA 4ftfA 4fzlA 4fzpA 4g0xA 4g9sB 4gaiA 4gbuA
4ggcA 4gmqA 4gofA 4gq4A 4gucA 4gxwA 4h4dA 4h4nA 4h6cA 4h7wA 4h8eA 4hddA 4he6A 4hi8B 4hlsA
4ht2A 4ht3B 4htgA 4hwvA 4i6yA 4ibyA 4ieuA 4iilA 4il7A 4ippA 4ipuA 4iqbA 4ivvA 4ix3A 4jb7A
4jf8A 4jg2A 4jglA 4jguA 4jhtA 4jiuA 4jm1A 4jp6A 4jtmA 4jxhA 4kdwA 4kn9S 4kt3B 4l2hA 4l2iA
4l6dA 4l9pB 4ld1A 4lerA 4lhsA 4ljhA 4lpqA 4lttA 4lupA 4m1vA 4m5rA 4mjdA 4muqA 4n2pA 4n4bA
4n6pA 4nbpA 4nkpA 4nzkA 4o0aA 4orlA0

b) List of 63 PDB Chains used for validation during early-stopped training.

1atgA 1f86A 1h12A 1k5nA 1lq9A 1mwqA 1oaiA 1qqfA 1t2dA 1vlyA 1wdpA 1xmkA 2aebA 2bt9A 2ciwA
2fb6A 2gqtA 2ia7A 2lisA 2oizA 2p8iA 2qvkA 2rffA 2vlaA 2wlvA 2xolA 2yhgA 3a72A 3b5mA 3buuA
3clmA 3exvA 3fcnA 3g91A 3h5jA 3hzpA 3ipjA 3ke7A 3lhnA 3mjfA 3nufA 3oheA 3plwA 3qnsA 3s44A
3swoA 3u2uA 3uueA 3vz9D 3zrxA 4alzA 4bn4A 4do4A 4esmA 4fh0A 4gc3A 4h6qA 4hwmA 4iumA 4jk8A
4l2iB 4m0kA 4nyqA

Supplementary Table S2

List of 434 protein chains with effective sequence counts used to benchmark MetaPSICOV on smaller sequence families.

3WU2D	3	3WU2E	7	1EERA	8	3Q7CA	8	2OIZD	9
4FMHA	9	2FHZB	10	1EEXB	10	4EGDA	10	1RZHM	10
1G73A	11	2INWA	12	3REAA	13	1OO0A	13	3WU2H	13
4DEYB	14	2JCQA	14	1KQRA	14	2P58B	14	3TUOA	14
4K12B	14	4L5RC	15	2HNGA	15	4J9YB	15	1IUQA	16
2GIBA	16	3TEQA	17	3UMHA	17	3KU3A	17	4BK0A	17
3U28B	18	3RNVA	18	1K8KF	18	1NKZA	18	1K04A	19
3LQ9A	19	3WU2O	20	4G92A	20	1N13A	20	2GRRB	20
2QZQA	20	1EEXG	20	3JSRA	20	3MSWA	20	2BO9B	20
4I1FA	21	1GVPA	21	3OFGA	21	3GP6A	21	1LMIA	22
4EW5A	22	3N6YA	22	4IX7A	22	3GQHA	22	2J9UB	23
3MXNB	23	3LT7A	23	4KOOA	23	3HMSA	24	2XGRA	24
3N8BA	24	4JPRA	24	1Q8DA	24	4BVQA	24	3OSTA	24
3THGA	25	2P14A	25	4I4TE	25	1W2YA	26	4K2PA	26
3WU2Z	26	1T61A	27	3UZQB	27	3C2QA	27	2PU9A	27
1C1YB	27	4KA9A	27	1FTRA	27	1BEAA	28	1Y96B	28
2QV6A	28	3T92A	28	2GUKA	29	2YGNA	29	2XUVA	29
2XOCA	29	1RZHH	30	3MXNA	30	3A8GA	30	4E6FA	30
1LKIA	30	3F4MA	30	4I9OA	31	2BBAA	32	4ATHA	32
4B2FA	32	1XKPA	32	2JDII	33	4JQUB	33	2Z3QA	33
3MCBB	33	4LDVA	33	3VOQA	34	3HWPA	34	3AQEA	34
4GIPA	35	2FKKA	35	4KMYA	36	1F3UB	36	3EA6A	36
2D1LA	36	4C0ZA	37	1MNNA	37	1K4NA	37	1XCRA	38
2HU9A	38	3IM6A	38	2P3PA	38	3TS9A	38	2J6GA	38
2NMLA	38	2Q9RA	38	4C2WC	39	3NRHA	39	3SC0A	39
1J98A	39	2OVSA	39	2GA1A	39	4HWCA	39	3HIEA	39
1BGCA	40	1EG3A	40	4CRUB	40	4KPIA	40	1R0DA	40
3C18A	40	4KQ7A	40	3A2ZA	40	4AQRD	41	2IA1A	41
2VSMB	41	2AP3A	41	3NJCA	42	3PS0A	42	2V6XB	42
1K8KE	42	3GE3E	42	3OBHA	43	1D8HA	43	2IM8A	43
4GIWA	43	3MMYB	43	3Q20A	43	1WPBA	44	3A77A	44
2PSPA	44	3USHA	44	1G8EA	44	3VP9A	45	1ROCA	45
4BQNA	45	2W56A	45	4KRDB	45	4H3KB	46	3DZAA	47
3CX5G	47	4ADNA	47	4BBOA	47	2I74A	47	3RF3A	47
4A6QA	48	2Y1BA	48	2F01A	48	1F00I	49	1MTYB	49
2J43A	49	4JHQA	50	2PU3A	50	2R4GA	51	4A2VA	51
3UV1A	51	1W53A	52	3HN5A	52	2QHQA	53	3TOWA	53
2YK4A	53	2REUA	53	2F5TX	54	4J8SA	54	3VHJA	55
3C9QA	55	3VZHA	55	3CBNA	55	3AG3H	56	2HUHA	56
3QYFA	56	3CX5F	56	4CCVA	56	4J32A	57	1I7WB	57
1YQEA	57	2BBRA	58	3NPHB	58	1NH2D	58	2XXPA	59
3AG3G	59	2P2VA	59	3D34A	59	3DGPB	61	1UPKA	61
2YV9A	62	3VBCA	63	2O62A	63	3NCEA	64	4GOQA	65
3NR5A	65	3SXUB	65	3U4GA	66	3CX5I	66	1IG0A	66
3V46A	67	2Y7LA	68	4H0AA	68	2X55A	68	3D33A	68
3Q3MB	69	2J6AA	70	4B89A	70	3LKMA	70	3F5RA	70
3GD0A	70	2D68A	70	1PX5A	70	1KZQA	70	1POCA	71
3NRXA	71	3RLSA	71	3BPJA	71	3W1EA	71	2Y3VA	72
4DDPA	72	1F3UA	72	1KCMA	72	2OKMA	73	3ZQOA	74
2SICI	74	2V7KA	74	4GIOA	74	2F4MB	75	1OEYA	76
2Q03A	77	4LKMA	78	4GDZA	78	4IOYX	78	4ATGA	78
3WP9A	79	1DZFA	79	4KJMA	79	2HJNA	79	2H1TA	79
3VGIA	79	3WX1A	80	4LUNU	80	2XPPA	81	3UD1A	81
1U7LA	82	2OEBA	82	3O2TA	82	3GKJA	82	2ERVA	83
3O6CA	83	4K98A	84	1ZD0A	84	3FHVA	84	1XODA	84
3CHHA	84	3MHSB	84	3HFTA	85	1WV3A	85	2OKFA	85
3U1CA	86	2ES4D	86	3C5NA	86	4AT7B	87	2P4FA	87
2VLQA	87	4LIMA	87	3LGBA	88	4E29A	88	2I9XA	89
2E7VA	91	2V94A	92	3QZMA	93	3NNBA	93	3BUTA	94
1SDIA	94	2IT2A	94	4L9HA	94	2WJRA	94	3H8TA	94
3D7AA	94	4FGQA	95	1R9WA	95	3DXTA	96	4BJIA	97
2ZX2A	97	3POWA	97	3PL0A	98	4G6TA	98	3FGRB	99
2QUPA	99	1JHSA	99	2P26A	100	4HZDA	100	1UTGA	100
2WNKA	100	3P0YA	100	3PR6A	101	3KMIA	101	3GNZP	101
4BWCA	104	3KDFA	104	3QF2A	105	4FQEA	105	3BHWA	105
3SBTB	109	2WVQA	109	2IMHA	109	3GAEA	109	2NZ7A	110
1TS9A	110	1JIDA	110	4JMDA	111	2FCOA	111	4CDPA	111
3ONJA	112	2GAKA	113	2NW8A	114	4HRVA	114	3WJCA	114
3LTIA	114	2XDJA	115	1JYOA	116	3T7HA	116	4E1SA	116
1MFWA	117	3QR7A	118	4IHZA	118	2CAYA	119	3TC2A	120
3FIDA	120	4DLHA	120	3HRGA	121	2F9HA	123	2O2KA	124

2V0PA	125	1F46A	126	3ZY2A	129	1RYLA	129	2FSUA	130
3UWSB	131	2I9WA	132	2BKYA	133	2BL0A	133	1NLQA	135
3G36A	136	2PW0A	137	2WZOA	137	2WNFA	138	4J7NA	139
3EVYA	140	3KEVA	140	4L0JA	141	2Y4YA	141	2PNWA	141
3QF7C	141	4I8IA	143	4CV4A	143	2A9IA	144	3JV1A	144
3ABDA	145	2ZYZB	146	4KK7A	146	4GJ4A	148	2ZOUA	149
3G3TA	150	3AKJA	150	2OX7A	151	4DQ9A	152	3V3LA	152
3FO5A	154	2J73A	155	1YLLA	160	1ZHVA	162	3OE3A	163
4LW9A	166	4I90A	169	2CWYA	170	4M8ZA	170	2Z14A	172
3CHMA	172	2G5GX	174	2PH0A	174	2ZCAA	176	1VBWA	178
4FQGA	178	3ZHFA	180	4FMWA	181	1OCYA	183	3ZLCA	192
2Y8YA	193	3TE8A	195	3N6ZA	196	1IZMA	200	1IWMA	200
3QDHA	205	2Z0JA	206	2X9ZA	207	1PV5A	208	3TUTA	211
4B60A	211	2AJ7A	226	1T9IA	231	2OQZA	241	1U0SA	246
1R0UA	246	3TIPA	259	2I5IA	263	4JJ2A	268	2X46A	276
2IDLA	293	2YMVA	294	2V05A	305	4K4KA	330		

Supplementary Table S3

Summary of input features used by MetaPSICOV.

	size	number of features
**STAGE 1**		**672**
WINDOWS		
two 9-residue windows at residues i,j	18	-
5-residue window at midpoint between i,j	5	-
COLUMN FEATURES		
amino acid composition	21	483
probability of a helix, strand and coil (PSIPRED)	3	69
predicted solvent accessibility (SOLVPRED)	1	23
Shannon entropy of the alignment column	1	23
missing data (e.g. if window exceeds the limits of the sequence)	1	23
COEVOLUTION FEATURES		
mutual information		1
normalised mutual information (Dunn et al., 2008)		1
mean contact potential		1
PSICOV score		1
mfDCA score		1
CCMpred score		1
sequence separation (|i-j|<5; |i-j|= {5, 6, …, 13}; 14≤|i-j|≤17; 18≤|i-j|≤22; 23≤|i-j|≤27; 28≤|i-j|≤37; 38≤|i-j|≤47; |i-j|≥48)		16
GLOBAL SEQUENCE FEATURES		
global amino acid composition (incl. gap)		21
global fractions of predicted secondary structures		3
global average predicted solvent exposure		1
log sequence length		1
log number of sequences in the alignment		1
log effective number of sequences		1
global average Shannon entropy		1
**STAGE 2**		**731**
WINDOWS		
two 11-residue windows at residues i,j	22	-
FEATURES		
column features (as in Stage 1)	27	594
sequence separation (as in Stage 1)		16
11x11 matrix of Stage 1 outputs		121

Supplementary Table S4

Mean precision values for the top-L, top-L/2, top-L/5 and top-L/1 contacts, L= length of target protein, at different sequence separation ranges i-j ≥ 5 and i-j ≥ 23, where the Cβ-Cβ distance < 8 Å. Each row corresponds to the first stage results from a different combination of the three coevolution methods, with all other neural network features included.

	[i-j]≥5				[i-j]≥23			
	L	L/2	L/5	L/10	L	L/2	L/5	L/10
PSICOV	0.39	0.43	0.55	0.7	0.29	0.37	0.45	0.52
DCA	0.45	0.5	0.67	0.81	0.36	0.47	0.6	0.68
PSICOV+DCA	0.49	0.57	0.76	0.87	0.41	0.54	0.7	0.78
CCMpred	0.6	0.75	0.88	0.94	0.44	0.59	0.75	0.82
PSICOV+CCMpred	0.63	0.78	0.89	0.95	0.46	0.61	0.77	0.84
DCA+CCMpred	0.65	0.79	0.9	0.95	0.47	0.62	0.77	0.84
PSICOV+DCA+CCMpred	0.66	0.79	0.9	0.95	0.47	0.63	0.78	0.85

Supplementary Table S5

Results for structure-based cross-validation of MetaPSICOV benchmark results. a) The subset of 101 proteins used to check cross-validation results. Any protein sharing a common domain superfamily according to CATH release 4.0 was excluded from the original set of 150 proteins. b) Superfamily filtered benchmark results for residue separation [i-j] ≥ 5. c) Results for [i-j] ≥ 23. In each case the mean precision is given, along with the sample standard deviation and calculated t-test p-value.

a)

1a3aA 1a6mA 1a70A 1aapA 1aoeA 1atzA 1avsA 1bdoA 1behA 1brfA
1c44A 1chdA 1ckeA 1ctfA 1cxyA 1d0qA 1d4oA 1dbxA 1dixA 1dlwA
1dmgA 1ej0A 1ej8A 1ek0A 1f6bA 1fcyA 1fk5A 1fvgA 1fx2A 1g2rA
1g9oA 1gbsA 1gmiA 1gmxA 1gz2A 1h0pA 1h2eA 1h4xA 1h98A 1hh8A
1htwA 1hxnA 1i1jA 1i1nA 1i4jA 1i58A 1i71A 1ihzA 1im5A 1j3aA
1jbkA 1jkxA 1jo0A 1jo8A 1jwqA 1jyhA 1k6kA 1k7cA 1k7jA 1kidA
1kq6A 1ktgA 1lm4A 1lpyA 1m4jA 1m8aA 1mk0A 1nb9A 1ne2A 1npsA
1nrvA 1ny1A 1o1zA 1p90A 1pchA 1qf9A 1qjpA 1ql0A 1roaA 1rw7A
1rybA 1svyA 1t8kA 1tifA 1tqgA 1tzvA 1vfyA 1vhuA 1vjkA 1vmbA
1whiA 1wjxA 1wkcA 1xdzA 1xffA 1xkrA 2arcA 2hs1A 2mhrA 3borA
3dqgA

b)

		L			L/2			L/5			L/10		
	N	Precision	StdDev	p	Precision	StdDev	p	Precision	StdDev	p	Precision	StdDev	p
All	150	0.71	0.114	0.49	0.83	0.099	0.43	0.91	0.086	1.00	0.94	0.078	0.30
No Sfams	101	0.68	0.114		0.8	0.1		0.91	0.083		0.95	0.074	

c)

		L			L/2			L/5			L/10		
	N	Precision	StdDev	p	Precision	StdDev	p	Precision	StdDev	p	Precision	StdDev	p
All	150	0.54	0.167	0.63	0.69	0.185	1.00	0.83	0.177	1.00	0.88	0.169	1.00
No Sfams	101	0.53	0.156		0.69	0.173		0.83	0.164		0.88	0.153	

Supplementary Table S6

TM scores for representative models extracted from FRAGFOLD ensembles for each of the 150 benchmark protein chains.

PDB Code & Chain	No contacts	PSICOV contacts	MP Stage1 contacts	MP Stage2 contacts
**1a3aA**	0.286	0.422	0.549	0.566
**1a6mA**	0.332	0.391	0.494	0.571
**1a70A**	0.279	0.353	0.400	0.424
**1aapA**	0.273	0.408	0.280	0.359
**1abaA**	0.278	0.477	0.527	0.599
**1ag6A**	0.224	0.351	0.460	0.453
**1aoeA**	0.176	0.305	0.244	0.334
**1atlA**	0.336	0.263	0.297	0.329
**1atzA**	0.293	0.578	0.381	0.404
**1avsA**	0.615	0.417	0.435	0.561
**1bdoA**	0.170	0.306	0.258	0.293
**1bebA**	0.424	0.248	0.440	0.506
**1behA**	0.191	0.236	0.278	0.249
**1bkrA**	0.314	0.415	0.534	0.578
**1brfA**	0.204	0.237	0.378	0.355
**1bsgA**	0.184	0.358	0.321	0.313
**1c44A**	0.265	0.241	0.456	0.434
**1c52A**	0.338	0.271	0.497	0.327
**1c9oA**	0.438	0.582	0.641	0.595
**1cc8A**	0.555	0.687	0.819	0.720
**1chdA**	0.225	0.530	0.581	0.525
**1cjwA**	0.242	0.394	0.572	0.502
**1ckeA**	0.247	0.394	0.402	0.400
**1ctfA**	0.402	0.561	0.509	0.636
**1cxyA**	0.278	0.463	0.451	0.473
**1cznA**	0.254	0.436	0.496	0.448
**1d0qA**	0.240	0.437	0.497	0.415
**1d1qA**	0.233	0.374	0.453	0.340
**1d4oA**	0.263	0.311	0.205	0.302
**1dbxA**	0.229	0.275	0.482	0.278
**1dixA**	0.291	0.204	0.260	0.304
**1dlwA**	0.588	0.614	0.519	0.625
**1dmgA**	0.225	0.285	0.316	0.336
**1dqgA**	0.186	0.206	0.243	0.251
**1dsxA**	0.269	0.353	0.437	0.379
**1eazA**	0.382	0.394	0.589	0.623
**1ej0A**	0.274	0.394	0.556	0.605
**1ej8A**	0.269	0.206	0.341	0.278
**1ek0A**	0.234	0.541	0.552	0.455
**1f6bA**	0.288	0.395	0.473	0.405
**1fcyA**	0.302	0.438	0.256	0.350
**1fk5A**	0.375	0.441	0.502	0.446
**1fl0A**	0.253	0.229	0.313	0.370
**1fnaA**	0.719	0.687	0.757	0.744
**1fqtA**	0.309	0.524	0.488	0.515
**1fvgA**	0.229	0.391	0.501	0.400
**1fvkA**	0.213	0.585	0.559	0.441
**1fx2A**	0.255	0.559	0.501	0.508
**1g2rA**	0.387	0.515	0.423	0.454
**1g9oA**	0.369	0.565	0.677	0.474
**1gbsA**	0.223	0.321	0.492	0.497
**1gmiA**	0.177	0.254	0.330	0.404
**1gmxA**	0.375	0.583	0.631	0.604
**1guuA**	0.405	0.645	0.510	0.522
**1gz2A**	0.246	0.303	0.278	0.287
**1gzcA**	0.211	0.200	0.298	0.263
**1h0pA**	0.213	0.264	0.287	0.252
**1h2eA**	0.227	0.436	0.465	0.410

PDB Code & Chain	No contacts	PSICOV contacts	MP Stage1 contacts	MP Stage2 contacts
**1h4xA**	0.340	0.559	0.653	0.733
**1h98A**	0.227	0.284	0.325	0.367
**1hdoA**	0.210	0.572	0.624	0.655
**1hfcA**	0.250	0.312	0.246	0.346
**1hh8A**	0.282	0.496	0.448	0.394
**1htwA**	0.272	0.511	0.671	0.583
**1hxnA**	0.261	0.189	0.261	0.297
**1i1jA**	0.220	0.178	0.261	0.353
**1i1nA**	0.322	0.538	0.438	0.467
**1i4jA**	0.358	0.413	0.621	0.487
**1i58A**	0.224	0.399	0.435	0.460
**1i5gA**	0.401	0.475	0.541	0.573
**1i71A**	0.246	0.257	0.184	0.322
**1ihzA**	0.291	0.535	0.493	0.441
**1iibA**	0.606	0.567	0.639	0.622
**1im5A**	0.278	0.506	0.530	0.464
**1iwdA**	0.213	0.438	0.470	0.398
**1j3aA**	0.287	0.345	0.504	0.460
**1jbeA**	0.639	0.811	0.798	0.811
**1jbkA**	0.219	0.222	0.293	0.250
**1jfuA**	0.294	0.416	0.578	0.464
**1jfxA**	0.211	0.534	0.585	0.548
**1jkxA**	0.305	0.413	0.578	0.461
**1jl1A**	0.278	0.408	0.385	0.414
**1jo0A**	0.421	0.562	0.612	0.486
**1jo8A**	0.814	0.796	0.839	0.799
**1josA**	0.295	0.426	0.632	0.611
**1jvwA**	0.206	0.332	0.400	0.428
**1jwqA**	0.292	0.486	0.459	0.528
**1jyhA**	0.233	0.292	0.369	0.294
**1k6kA**	0.280	0.531	0.604	0.605
**1k7cA**	0.200	0.448	0.402	0.419
**1k7jA**	0.185	0.286	0.260	0.307
**1kidA**	0.265	0.417	0.377	0.299
**1kq6A**	0.281	0.377	0.516	0.400
**1kqrA**	0.205	0.186	0.190	0.232
**1ktgA**	0.270	0.304	0.360	0.250
**1ku3A**	0.514	0.607	0.546	0.469
**1kw4A**	0.538	0.505	0.652	0.645
**1lm4A**	0.203	0.368	0.407	0.259
**1lo7A**	0.229	0.458	0.570	0.498
**1lpyA**	0.224	0.483	0.368	0.297
**1m4jA**	0.260	0.251	0.502	0.338
**1m8aA**	0.432	0.475	0.305	0.491
**1mk0A**	0.301	0.266	0.315	0.373
**1mugA**	0.286	0.310	0.301	0.303
**1nb9A**	0.214	0.359	0.408	0.335
**1ne2A**	0.309	0.392	0.536	0.401
**1npsA**	0.291	0.362	0.330	0.366
**1nrvA**	0.467	0.550	0.461	0.580
**1ny1A**	0.236	0.314	0.437	0.379
**1o1zA**	0.383	0.498	0.597	0.597
**1p90A**	0.264	0.269	0.498	0.435
**1pchA**	0.303	0.504	0.617	0.663
**1pkoA**	0.267	0.322	0.349	0.295
**1qf9A**	0.263	0.447	0.385	0.415
**1qjpA**	0.189	0.276	0.279	0.282
**1ql0A**	0.194	0.243	0.306	0.288
**1r26A**	0.474	0.687	0.698	0.634
**1roaA**	0.395	0.425	0.453	0.418
**1rw1A**	0.292	0.419	0.578	0.448
**1rw7A**	0.206	0.355	0.461	0.442
**1rybA**	0.290	0.369	0.369	0.262
**1smxA**	0.316	0.395	0.375	0.375
**1svyA**	0.426	0.527	0.524	0.599
**1t8kA**	0.441	0.675	0.728	0.673
**1tifA**	0.325	0.480	0.541	0.416

PDB Code & Chain	No contacts	PSICOV contacts	MP Stage1 contacts	MP Stage2 contacts
**1tqgA**	0.389	0.663	0.738	0.822
**1tqhA**	0.207	0.358	0.471	0.531
**1tzvA**	0.314	0.693	0.739	0.731
**1vfyA**	0.284	0.218	0.358	0.287
**1vhuA**	0.412	0.589	0.653	0.628
**1vjkA**	0.338	0.510	0.524	0.502
**1vmbA**	0.263	0.439	0.524	0.290
**1vp6A**	0.295	0.251	0.470	0.385
**1w0hA**	0.211	0.334	0.405	0.581
**1whiA**	0.241	0.335	0.373	0.359
**1wjxA**	0.208	0.269	0.371	0.414
**1wkcA**	0.244	0.470	0.536	0.566
**1xdzA**	0.267	0.574	0.641	0.513
**1xffA**	0.221	0.622	0.442	0.379
**1xkrA**	0.258	0.268	0.249	0.270
**2arcA**	0.203	0.320	0.264	0.298
**2cuaA**	0.264	0.440	0.410	0.442
**2hs1A**	0.224	0.218	0.220	0.231
**2mhrA**	0.416	0.586	0.643	0.675
**2phyA**	0.337	0.411	0.570	0.323
**2tpsA**	0.312	0.521	0.540	0.479
**2vxnA**	0.225	0.648	0.661	0.570
**3borA**	0.247	0.620	0.610	0.561
**3dqgA**	0.249	0.247	0.359	0.415
**5ptpA**	0.205	0.350	0.462	0.516

Supplementary Figure S1

Mean precision values for protein families with small numbers of effective sequences. L= length of target protein, at sequence separation ranges i-j ≥ 5 (all contacts) and i-j ≥ 23 (long range).

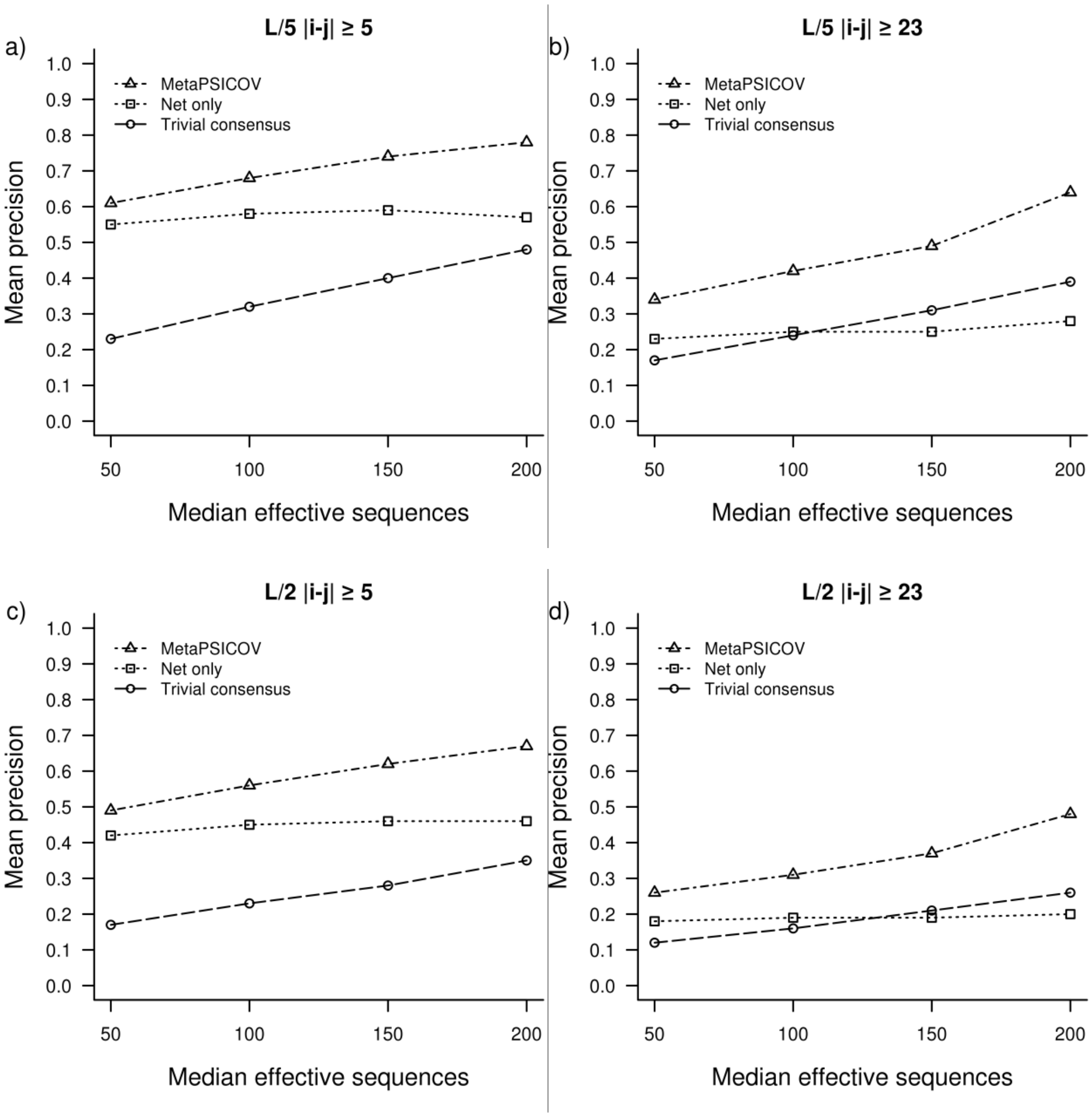

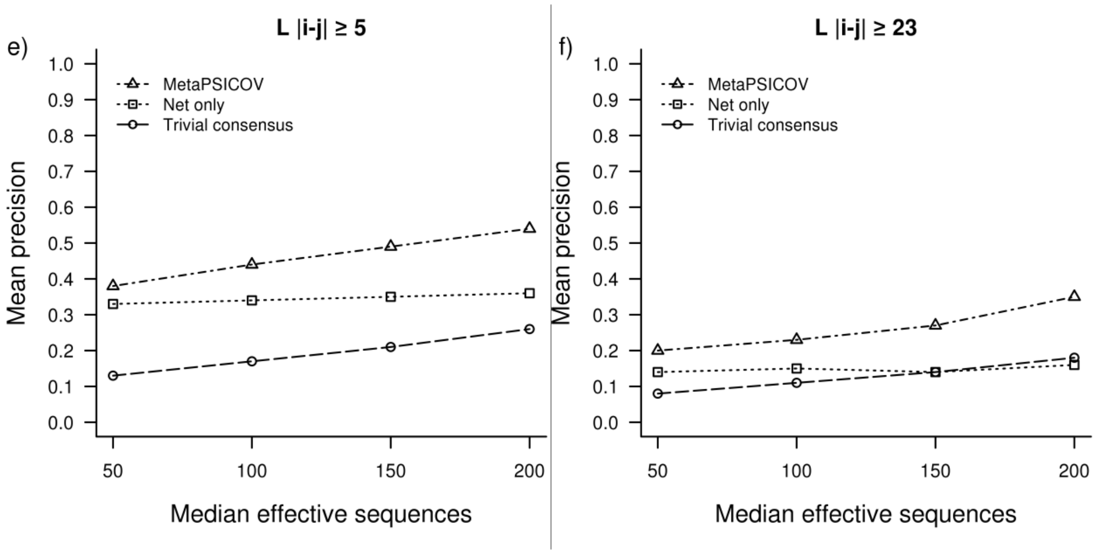
e)
L |i-j| ≥ 5
MetaPSICOV
Net only
Trivial consensus
Mean precision
Median effective sequences
1.0
0.9
0.8
0.7
0.6
0.5
0.4
0.3
0.2
0.1
0.0
50
100
150
200
f)
L |i-j| ≥ 23
MetaPSICOV
Net only
Trivial consensus
Mean precision
Median effective sequences
1.0
0.9
0.8
0.7
0.6
0.5
0.4
0.3
0.2
0.1
0.0
50
100
150
200

Supplementary Figure S2

Comparison of model TM-score for FRAGFOLD generated models using contacts from a) MetaPSICOV stage 1 and PSICOV b) MetaPSICOV stage 2 and PSICOV c) MetaPSICOV stage 1 and MetaPSICOV stage 2 (line x=y shown for reference).

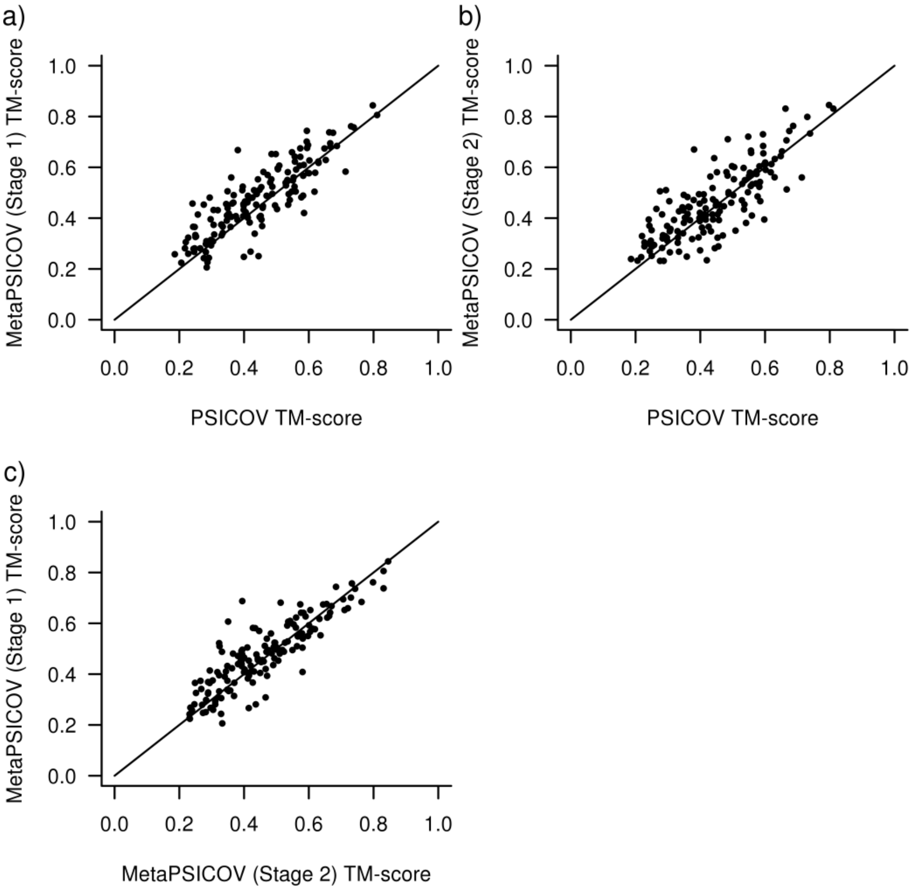

Supplement: Supplementary Data [file supp_btu791_btu791-metapsicov_supplementary_v1_0.pdf]
